# *Aedes albopictus* is a competent vector of Zika virus: A meta-analysis

**DOI:** 10.1371/journal.pone.0216794

**Published:** 2019-05-21

**Authors:** Benjamin A. McKenzie, Alan E. Wilson, Sarah Zohdy

**Affiliations:** 1 School of Forestry and Wildlife Sciences, Auburn University, Auburn, Alabama, United States of America; 2 School of Fisheries, Aquaculture, and Aquatic Sciences, Auburn University, Auburn, Alabama, United States of America; 3 College of Veterinary Medicine, Auburn University, Auburn, Alabama, United States of America; Centro de Pesquisas René Rachou, BRAZIL

## Abstract

**Background:**

Much work has been done in recent years to determine the vector competence of *Aedes albopictus* (Skuse) for Zika virus (ZIKV). If competent, *Ae*. *albopictus* could become an important vector in the spread of ZIKV to areas which until now have been considered safe from the virus. Despite much speculation about *Ae*. *albopictus’* competence for ZIKV, there have been, to date, no quantitative syntheses of *Ae*. *albopictus’* competence, nor have the potentially confounding differences between studies been addressed.

**Methodology/ principle findings:**

This study represents a quantitative meta-analysis of the literature surrounding this topic by examining infection rates (IR) and transmission rates (TR) among sample populations of *Ae*. *albopictus* at 7 and 14 days post infection (dpi) across 15 journal articles comprising 23 studies. Our analyses examined potentially confounding variables in the studies contained therein, including: geographic origin of viral strain or mosquito population tested, whether sympatry of the tested viral strain and mosquito population was important, and freshness of blood meal. Our results suggest 1) *Ae albopictus* is a competent vector for ZIKV and 2) that origin of *Ae*. *albopictus* population and origin of viral strain had significant effects on the competence of *Ae*. *albopictus* to transmit ZIKV.

**Conclusions/ significance:**

These results indicate a need to further explore the effects of methodology on vector competence studies and to examine in more detail the geographic variation in the competence of *Ae*. *albopictus* for ZIKV as well as the underlying causes of said variation. The ability of *Ae*. *albopictus* to carry and transmit ZIKV also points to a need to create new vector control strategies in case of a ZIKV outbreak in an area where *Ae*. *albopictus* is prominent. Finally, this study represents a potential template for future meta-analyses in the field of vector competence, where this type of study has been under-utilized despite the abundance of relevant studies.

## Introduction

Zika virus (ZIKV) is a mosquito-borne virus from the family *Flavaviridae*, genus *Flavivirus*, a genus which also contains dengue fever (DENV), West Nile (WNV), Japanese encephalitis (JEV), and yellow fever (YFV) viruses [1]. ZIKV was first isolated in 1947 from a sentinel rhesus monkey (*Macaca mulatta*) in the Zika forest of Uganda [2]. Over the next 60 years, ZIKV was detected several times in serological studies and routine arthropod-borne viruses (or arboviruses) surveillance across Africa and Southeast Asia [3–6]. In 2007, the first large ZIKV outbreak among humans occurred on Yap Island, in the Federated States of Micronesia [7,8]. Nearly 73% of the population op the island was infected during this outbreak [7]. Another outbreak which began French Polynesia in 2013 and spread across the pacific region further raised concerns because of the number of people infected and because ZIKV infection was linked in several cases to an autoimmune disease known as Guillaine-Barré Syndrome (GBS), which causes acute or subacute flaccid paralysis [9–11]. This outbreak of the Asian lineage of ZIKV spread to the Americas in 2015, infecting around a million citizens in Brazil alone, and spread to a total of 50 countries and territories by 2017 [12]. Along with GBS, this recent outbreak has also been linked to microcephaly in infants [13–15] and has shown potential for sexual transmission [16–19] making ZIKV an emerging infectious disease of high concern.

Many mosquito species have been implicated as potential vectors of ZIKV. The sylvatic vector of ZIKV is thought to be *Aedes africanus* [2], while *Aedes henselli* was implicated in the Yap Island outbreak [7,8,20], but the primary vector of the outbreak in the Americas, the Pacific region and Asia is thought to be *Aedes aegypti* [10,21], which has demonstrated competence for ZIKV in multiple laboratory tests [22]. Vector competence, in this case, meaning the ability to be infected by and to transmit a pathogen [23–25]. In contrast, most other *Aedes* species have shown potential for infection by ZIKV, but low potential for transmission [26,27]. *Ae*. *aegypti* is a medically important vector as it has a broad distribution, lives largely in urban areas, and has anthropophilic feeding tendencies [28,29]. Along with ZIKV, *Ae*. *aegypti* is considered the primary vector of DENV, urban YFV, and chikungunya fever virus (CHIKV) in most parts of the world [30]. A few studies have suggested *Culex quinquefasciatus*, a known vector of WNV, as a potential vector of ZIKV [31,32]. However, *Cx*. *quinquefasciatus* has largely been discounted as a vector of ZIKV through experimentation and critical review[26,33]. Currently, much of the debate in the field surrounds the species *Aedes albopictus*, which has long been considered the secondary vector of ZIKV even before its competence was experimentally assessed [34–36]. In the past decade or so, *Ae*. *albopictus*’ role as a vector for arboviruses has become more widely recognized [37]. *Ae*. *albopictus* is already widely considered a competent vector for 26 arboviruses, including viruses from the families *Flaviviridae* (e.g. DENV, YFV, etc.), *Togaviridae* (e.g. CHIKV, etc.) and *Bunyaviridae* (e.g. La Crosse virus, or LCV, etc.) [37–40]. In some parts of the world *Ae*. *albopictus* is even considered to be the primary vector of DENV and CHIKV [41–45].

If competent to carry and transmit ZIKV, *Ae*. *albopictus* represents a major new threat in the global transmission of the disease [34,35]. From the 1980s-2000s, *Ae*. *albopictus* spread from its native Asian range to occupy a nearly global distribution [46], making it one of the most invasive mosquito species in the world. *Ae*. *albopictus’* current range stretches across every continent but Antarctica, and it is continuing to spread further into North America and Europe [29,46]. Thus, if *Ae*. *albopictus* mosquitoes prove to be a competent vector for ZIKV world-wide, it may expand the ZIKV threat in more temperate countries once considered safe due to the absence of *Ae*. *aegypti* [34,35]. This means control strategies designed to limit the spread of ZIKV must be broader than if *Ae*. *aegypti* is the sole vector [47]. It must also be considered that vector competence may vary by mosquito population and viral strain, and that not all strains of ZIKV are thought to have the same epidemic potential [48–50]. Understanding the connections between vector competence and virus/ vector phylogeny may help to further focus control strategies and public health efforts.

While it is thought that *Ae*. *albopictus* may be a vector of ZIKV, at least in some parts of the world [22,51], there have been no meta-analyses of *Ae*. *albopictus’* competence for ZIKV. It is especially important to understand how competence varies across studies, as this may give us insight into potential geographical differences in *Ae*. *albopictus* ability to carry and transmit ZIKV, which is thought to be variable [22,51–54], as well as giving us a chance to examine other potential variables in the literature. Studies to date of *Ae*. *albopictus’* competence for ZIKV vary in methods, mosquito strains, and viral strains among other things. All of these variables are known to impact mosquitoes’ vector competence for DENV, CHIKV and other flaviviruses, and it seems likely that they would affect competence for ZIKV as well [22,43,44]. The variation in vector competence among different mosquito populations/viral strains may be due to micro-adaptations in the host-pathogen genome, which could mean co-occurrence of mosquito population and ZIKV strain could affect competence of populations for arboviruses [48]. Laboratory-based infection methods can also cause variation in vector competence results. Richards et al., 2007 [55] and Ciota et al., 2017 [56] suggest that freezing viral blood meals before allowing mosquitoes to feed on them can cause dramatic underestimates of vector competence. Meanwhile, Roundy et al., 2017 [24] suggests that mosquitoes fed on live mice demonstrate higher vector competence than those fed on artificial blood meals. It is important to understand how different methods affect the findings of *Ae*. *albopictus’* competence for ZIKV.

The objectives of this study were to: 1) determine the overall vector competence of *Ae*. *albopictus* for ZIKV and 2) examine the variation in the literature and the effects of geography and methodology on *Ae*. *albopictus’* competence for ZIKV. Specifically, whether geographic origin of viral strain, geographic origin of mosquito test population, sympatric co-occurrence of mosquito population and viral strain, freshness of blood-meal, viral titer (or dose) of blood-meal, and whether the blood-meal was artificial or taken from a live, murine source, have significant effects on *Ae*. *albopictus* vector competence for ZIKV.

## Methods

### Meta-analysis for vector competence

Despite the widespread use of meta-analyses as a tool in both the medical and ecological fields [57,58], surprisingly, and with a few notable exceptions [59,60], it has not been widely used in synthesizing vector competence research. Here, a meta-analysis of the literature surrounding *Ae*. *albopictus’* competence for ZIKV was used to address objectives associated with determining vector competence, infection rate (IR), and transmission rate (TR) among *Ae*. *albopictus*’ groups subjected to infection by ZIKV. For our purposes, IR is defined as the proportion of the test population of *Ae*. *albopictus* with positive titers of ZIKV in their midguts and TR is defined as the proportion of infected *Ae*. *albopictus* individuals with positive titers of ZIKV in their salivary glands or saliva. Since these rates vary throughout the incubation time of a virus, IR and TR were used at both 7 and 14 dpi, the most common windows of time at which these metrics are examined.

#### Identification of studies and inclusion criteria

This study was conducted in accordance with the Preferred Reporting Items for Systematic Reviews and Meta-Analysis (PRISMA) 2018 Guidelines [61]. Relevant studies were identified using searches of keywords through Clarivate Analytics’ Web of Science (http://webofknowledge.com/WOS_GeneralSearch) and PubMed (http://www.ncbi.nlm.nih.gov/pubmed/) search engines. The search was refined to include only papers published in English between January 2014 and March 2018. The key terms used were as follows: Aedes albopictus, vector, competenc*, transmiss*, dissem*, infect*, zik*. Results were screened initially excluding all studies that did not include *Ae*. *albopictus* as a study organism. With the study objective to quantify *Ae*. *albopictus’* competence for ZIKV, results were further refined to include only primary, experimental vector competence studies and exclude field studies. Furthermore, only studies that assessed both IR and TR were included. Since vector competence varies a great deal over the infection period, all papers that did not examine competence at 7 and/or 14 dpi (the most commonly reported sampling times) were excluded. Studies in which mosquitoes were infected with a viral titer of 1 x 10^7^ ffu/pfu/TCID^50^ or lower were also excluded because they were significantly below the ID_50_ of *Ae*. *albopictus* for ZIKV as found by Ciota et al., 2017 [56]. A few papers (Azar et al. 2017, in particular) reported multiple IRs and TRs from separate experiments that used different methods, therefore these effect sizes (i.e. standardized numbers to measure the relationship between two variables) were listed as separate studies in our analyses.

**Fig 1:**
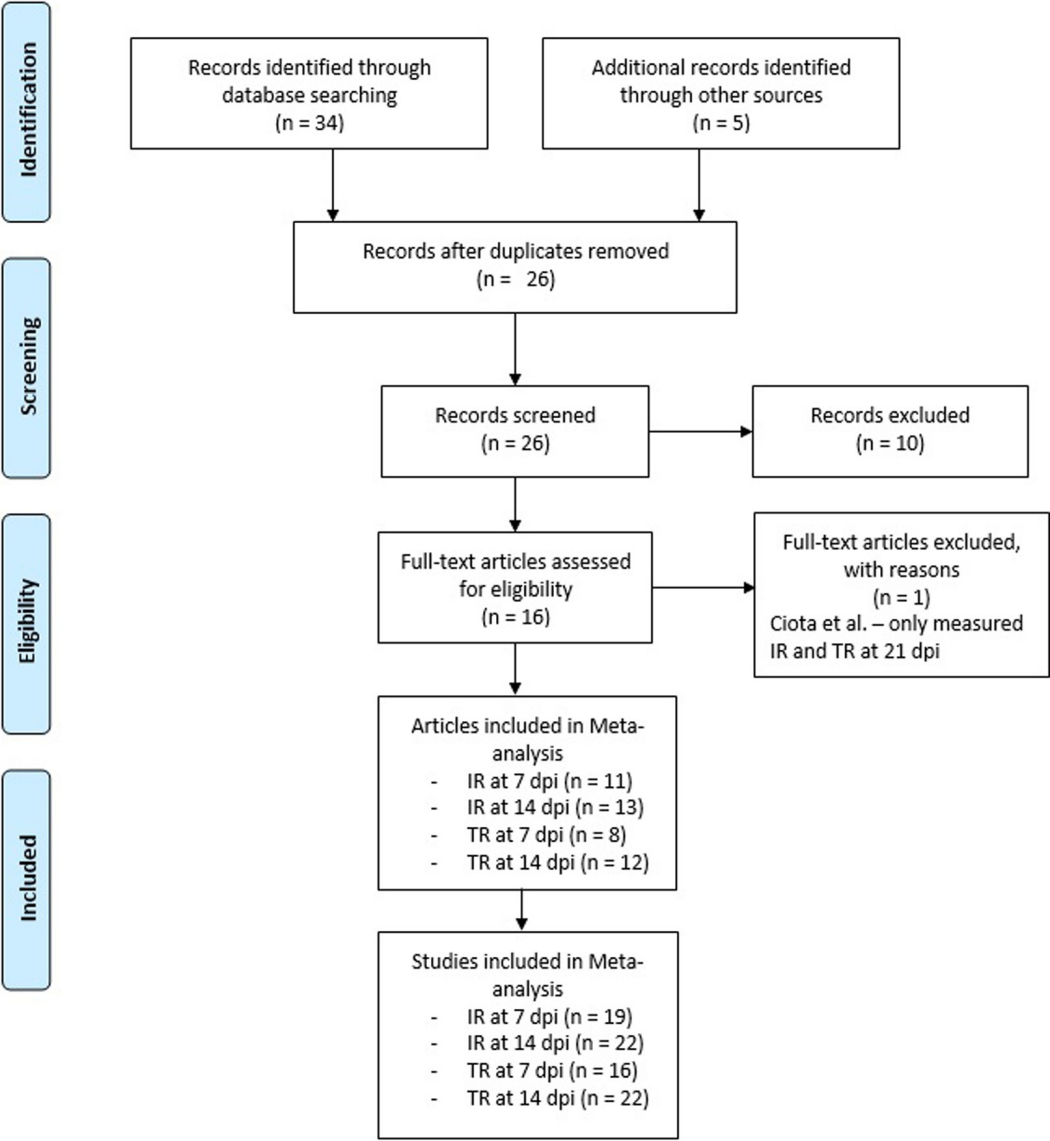
The inclusion process for the meta-analysis conducted followed PRISMA guidelines. This meta-analysis was carried out and reported according to PRISMA guidelines [61]. Search of the databases returned 34 records, while 2 additional records were identified through a search of the literature and 3 more through later search of the literature. After all duplicates were removed 26 records remained, of which 10 were excluded because they were not relevant to this study. 1 article was excluded because they did not meet the criteria for inclusion. The remaining 15 articles were divided into multiple studies based on the number of separate effect sizes reported and included in different parts of the meta-analysis, depending on which metrics they reported (IR at 7 dpi, IR at 14 dpi, TR at 7 dpi, TR at 14 dpi).

Of the 34 results originally identified by the search criteria, 13 were removed as duplicates. Two additional papers [62,63] were identified through a search of the literature. Two more papers [64,65], were identified in October of 2018, following the original analysis, using the same keyword search in Google Scholar and refining the search to papers published in 2018. Of the remaining 26 studies screened for eligibility, 10 were excluded, either because they did not include *Aedes albopictus* as a study species or because they did not study competence for ZIKV (Fig 1). Ciota et al., 2017 [50] was excluded because it only reported IR and TR after 21 dpi. This left a total of 14 papers for the analysis [23,62–74]. Two papers, Heitmann et al., 2017 [66] and Azar et al., 2017 [56] assessed multiple populations in their papers using different methods, so data from these papers were split into separate “studies” (experiments using different methodologies, such as viral dosage, or different viral strain/ vector population combinations, and thus useful for understanding the effects of methodological moderators) and tested separately. A full list of studies long with their characteristics, and the models in which they were included can be found in S1 Table. Ten studies from Azar et al., 2017 [56] were excluded because they studied mosquitoes infected with viral titers lower than 1 x 10^7^ ffu/pfu/TCID^50^. This left a total of 23 studies. A list of which studies were used in which models can be found in the S1 Table.

### Data extraction

The following study characteristics from the research articles matching inclusion criteria were extracted by a single reviewer and entered into a database: IR at 7 dpi, IR at 14 dpi, TR at 7 dpi, TR at 14 dpi, origin of viral strain, origin of *Ae*. *albopictus* test population strain, whether infected blood-meal was given to mosquitoes fresh or after having been frozen, log transformed viral titer of blood-meal, and whether the blood-meal was given artificially or taken from a live, murine source. IR and TR were taken directly from tables or extracted from figures using the R package “metaDigitise” [75]. Variation exists between studies in reporting transmission rates, with some reporting TR as the proportion of transmitting mosquitoes in the whole test population instead of the infected population. All data not conforming to our definitions were transformed using information on sample size found in the papers.

### Statistical analysis

Because IR and TR are easily comparable between vector competence studies and are inherently meaningful measurements, these raw proportions were used as effect sizes in this study. Data were compiled into a spreadsheet and analyzed using the R package “metafor” [76]. A mixed-effects restricted maximum likelihood (REML) model was used to determine pooled estimates of IR and TR at both 7 and 14 dpi, using sample size to weight estimates. Since not all studies examined IR and TR at both 7 dpi and 14 dpi, not every study was included in every model. Heterogeneity of IR and TR across studies were assessed using a Cochran Q test (P < 0.05 is considered to indicate statistically significant heterogeneity). Because they are easy to analyze and widely understood and utilized, funnel plots were used to look for publication bias, while Egger’s method was used to test for asymmetry. Results of tests for asymmetry can be found in the S1 Fig. Models were rerun using study characteristics thought to affect outcome (i.e., geographic origin of viral strain, geographic origin of mosquito test population, geographic co-occurrence of mosquito population and viral strain, freshness of blood-meal, viral titer (or dose) of blood-meal, and whether the blood-meal was artificial or taken from a live, murine source) as moderators. We also tested for collinearity among our moderators using linear regression, finding a strong collinearity between artificial or live blood meal and freshness of bloodmeal. We determined these to be confounding moderators, as models with one moderator removed artificially inflated the effect of the other. The R code for models can be found in the S1 Table.

## Results

**Fig 2:**
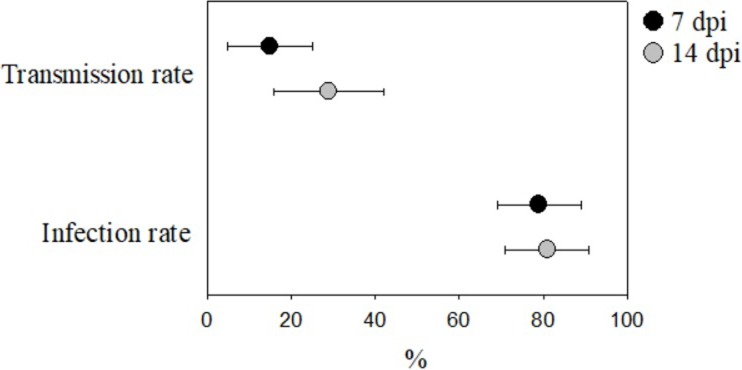
Mean infection rate and transmission rate (± 95% CI) of *Ae*. *albopictus* for ZIKV at 7 and 14 dpi. *Ae*. *albopictus* shows high infection rates at both 7 dpi (IR = 0.79, 95% CI = 0.69–0.81) and 14 dpi (IR = 0.81, 95% CI = 0.72–0.90). Transmission rates were considerably lower at both 7 dpi (TR = 0.15, 95% CI = 0.05–0.24), and 14 dpi (TR = 0.29, 95% CI = 0.16–0.42).

The pooled estimated IR of *Ae*. *albopictus* was 0.79 (95% CI 0.69–0.89) at 7 dpi and 0.81 (95% CI 0.72–0.90) at 14 dpi (Fig 2). Significant heterogeneity in IR estimates at both 7 and 14 dpi were observed with an *I*^*2*^ (Cochran’s Q test) of 93.6% (P < 0.0001) and 95.1% (P < 0.0001), respectively. For TR, significantly heterogeneous pooled estimates at 7 dpi of 0.15 (Fig 2; 95% CI 0.05–0.24; *I*^*2*^ = 90.3%; P < 0.0001) and at 14 dpi of 0.29 (Fig 2; 95% CI 0.16–0.42; *I*^*2*^ = 94.9%; P < 0.0001) were detected.

**Fig 3:**
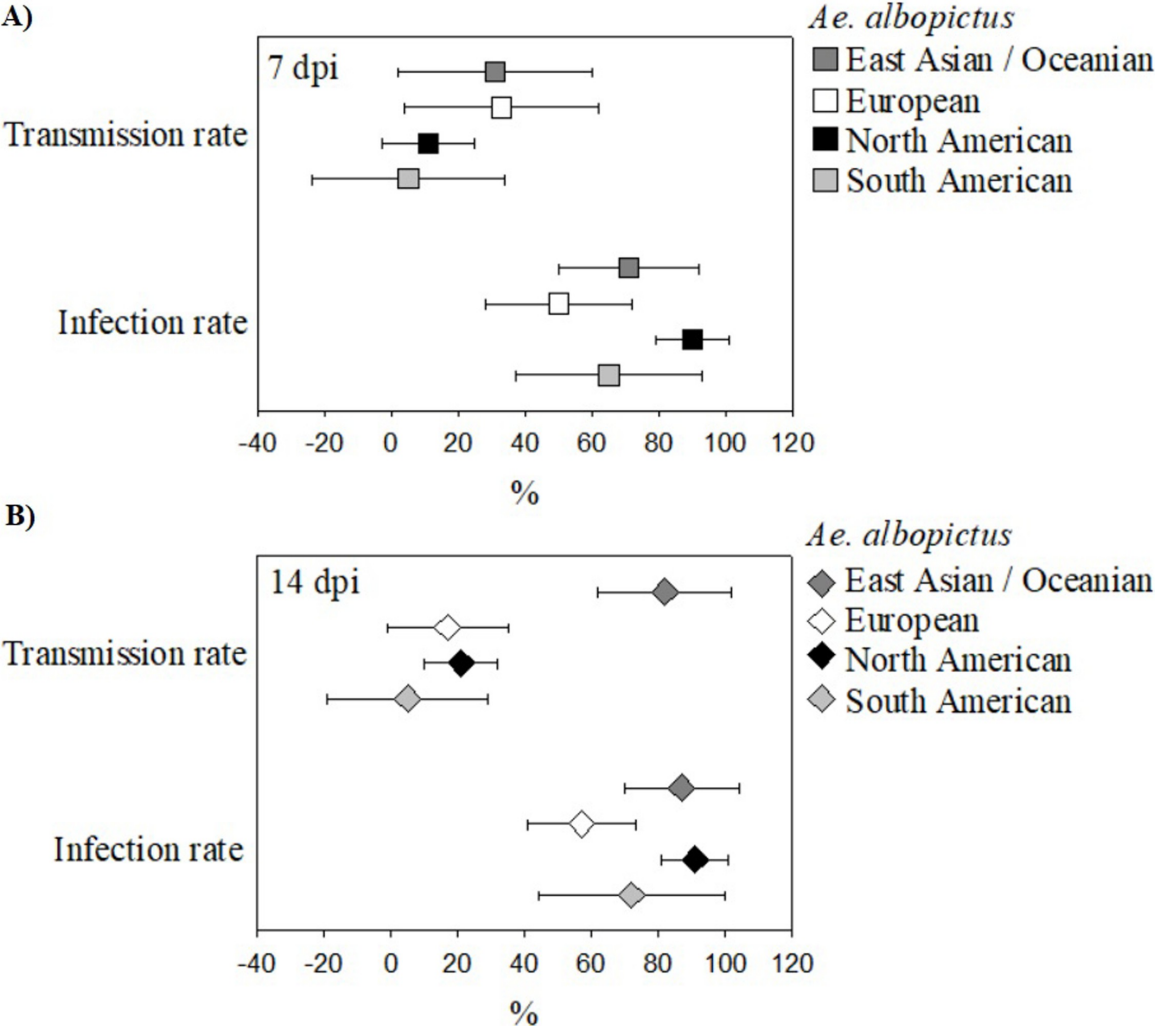
**Scatter plot showing mean IR and TR for ZIKV (± 95% CI) of geographically separated populations of *Ae*. *albopictus* at 7 (A) and 14 (B) dpi.** East Asian/ Oceanic and North American *Ae*. *albopictus* demonstrated higher IRs than other *Ae*. *albopictus* populations at both 7 dpi (IRs: East Asian/ Oceanic = 0.71, 95% CI = 0.50–0.92; European = 0.50, 95% CI = 0.28–0.72; North American = 0.90, 95% CI = 0.80–1.00; South American = 0.65, 95% CI = 0.37–0.93) and 14 dpi (IRs: East Asian/ Oceanic = 0.87, 95% CI = 0.70–1.04; European = 0.57, 95% CI = 0.41–0.74; North American = 0.91, 95% CI = 0.81–1.01; South American = 0.65, 95% CI = 0.37–0.93) though these results were not statistically significant. East Asian/ Oceanic and European *Ae*. *albopictus* showed the highest TRs at 7dpi (TRs: East Asian/ Oceanic = 0.31, 95% CI = 0.01–0.60; European = 0.33, 95% CI = 0.05–0.62; North American = 0.11, 95% CI = -0.03–0.24; South American = 0.05, 95% CI = -0.23–0.34) while East Asian/ Oceanic *Ae*. *albopictus* showed significantly higher TRs than other strains at 14dpi (TRs: East Asian/ Oceanic = 0.82, 95% CI = 0.61–1.03; European = 0.17, 95% CI = -0.01–0.35; North American = 0.21, 95% CI = 0.10–0.31; South American = 0.05, 95% CI = -0.19–0.28).

**Fig 4:**
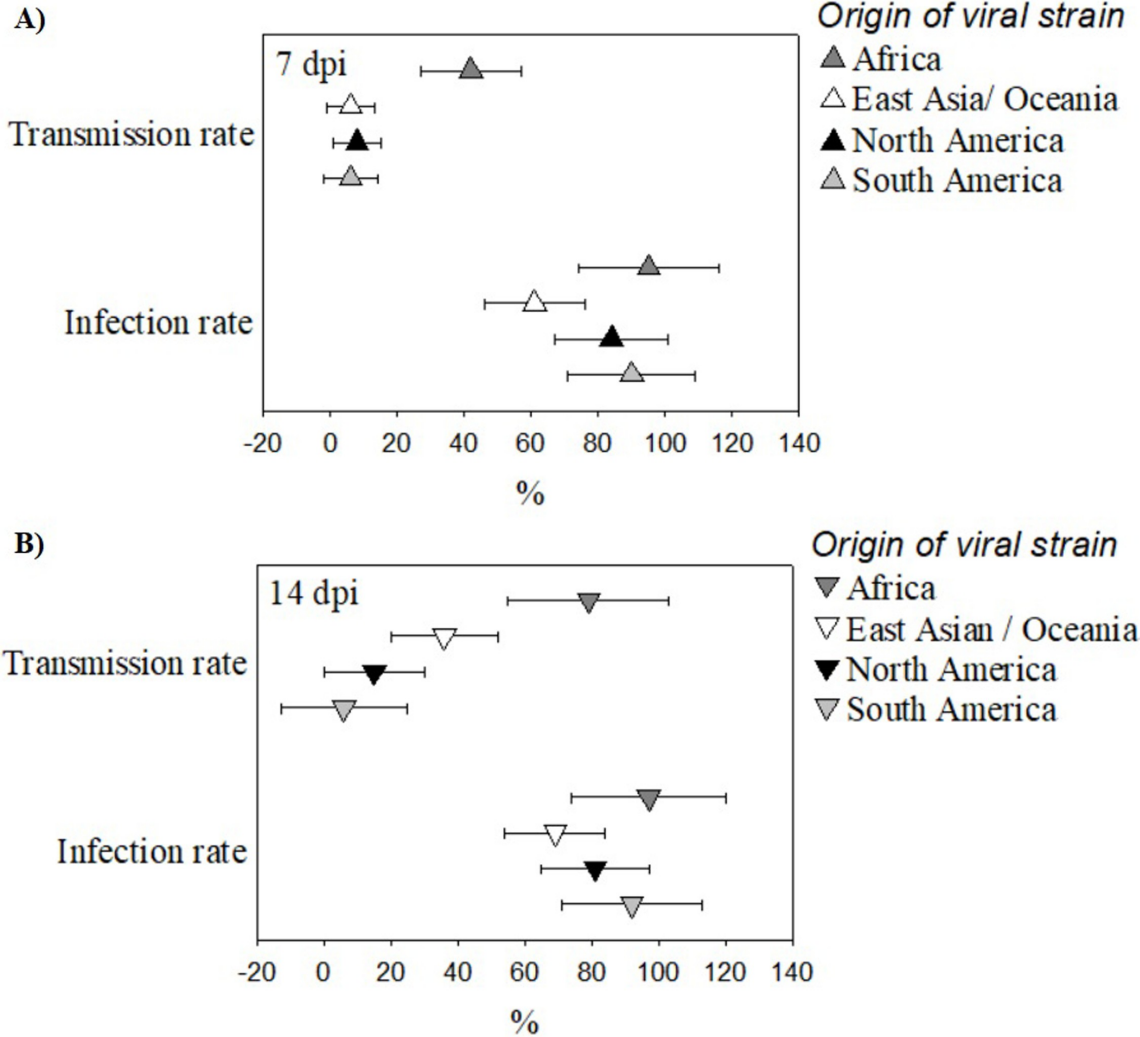
**Scatter plot showing mean IR and TR (± 95% CI) of *Ae*. *albopictus* infected with geographically separated strains of ZIKV at 7 (A) and 14 (B) dpi.** African and South American strains of ZIKV demonstrated significantly higher IRs than other ZIKV strains at both 7 dpi (IRs: African = 0.95, 95% CI = 0.74–1.17; East Asian/ Oceanic = 0.61, 95% CI = 0.46–0.76; North American = 0.84, 95% CI = 0.67–1.01; South American = 0.90, 95% CI = 0.71–1.10) and 14 dpi (IRs: African = 0.97, 95% CI = 0.73–1.2; East Asian/ Oceanic = 0.69, 95% CI = 0.54–0.84; North American = 0.81, 95% CI = 0.66–0.97; South American = 0.92, 95% CI = 0.72–1.13). African strains of ZIKV also demonstrated the highest TRs at 7 dpi (TRs: African = 0.42, 95% CI = 0.26–0.57; East Asian/ Oceanic = 0.06, 95% CI = -0.01–0.13; North American = 0.08, 95% CI = 0.01–0.14; South American = 0.06, 95% CI = -0.02–0.13) while African and East Asian/ Oceanic strains of ZIKV demonstrated the highest TRs at 14 dpi (TRs: African = 0.79, 95% CI = 0.56–1.03; East Asian/ Oceanic = 0.36, 95% CI = 0.20–0.52; North American = 0.15, 95% CI = 0.01–0.30; South American = 0.06, 95% CI = -0.13–0.25).

Tests of all moderators showed significant effects only on TR at 14 dpi (*Q* = 260.1, P < 0.0001). No significant moderator effects were found on IR at 7 dpi (*Q* = 13.1, P = 0.22) and 14 dpi (*Q* = 15.6, P = 0.11) or TR at 7 dpi (*Q* = 10.0, P = 0.44). Among moderators, geographic origin of *Ae*. *albopictus* test populations had significant TR at 14 dpi (Fig 3), as did origin of viral strain (Fig 4). Using mosquitoes from East Asia/Oceania as a reference group, models show that European, North American and South American mosquitoes displayed significantly lower TRs 14 dpi (P = 0.0025, P = 0.0002, P = 0.0013). Origin of viral strain also had significant effects on TR at 14 dpi (Fig 4), with East Asian/Oceanic, North American and South American viral strains showing significantly lower TRs than the reference African strains (P = 0.0079, P < 0.0017, P < 0.0001). Finally, freshness of blood meal, artificial or murine source of blood-meal, log viral dosage, and co-occurrence of mosquito population and viral strain had no significant effects on IR or TR at any stage.

## Discussion

This meta-analysis assessed 14 papers that accounted for 23 studies to determine the suitability of an invasive Asian mosquito species, *Ae*. *albopictus*, that is found on all continents except Antarctica, as a competent vector to both carry and transmit ZIKV. Across the studies analyzed, *Ae*. *albopictus* is a competent vector to both carry and transmit ZIKV in laboratory settings. Infection rates of ZIKV in *Ae*. *albopictus* at both 7 dpi (IR = 0.80; 95% CI 0.70–0.92) and 14 dpi (IR = 0.83; 95% CI 0.72–0.92) are high, on par with those found in similar studies of *Ae*. *aegypti* [24]. Transmission rates for ZIKV in this mosquito at 7 dpi (TR = 0.15; 95% CI 0.05–0.26) and 14 dpi (TR = 0.3; 95% CI 0.16–0.45) are low compared to *Ae*. *albopictus’* competence for other RNA viruses [40,77], but are still similar to numbers reported in many studies that examined the competence of *Ae*. *aegypti* for ZIKV [22].

Studies have suggested that geographically disparate populations of *Ae*. *aegypti* vary in their competence for ZIKV [24,67]. This meta-analysis suggests that *Ae*. *albopictus’* competence for ZIKV varies geographically as well. The reason for geographic variation in vector competence of *Ae*. *aegypti* and *Ae*. *albopictus* for ZIKV has been hypothesized to be a barrier that prevents infection of epithelial cells in the midgut [52,56,67,68] and/ or prevent transmission of ZIKV from the salivary gland to a host [53,64]. These barriers may be related to mosquitoes’ microbiome [78,79] or to interactionsbetween viral and mosquito genomes, which may also drive mosquito competence for a multitude of arboviruses, including ZIKV [48,80]. Since vector competence is thought to be influenced by regional micro-adaptions in the genome [48,80], a significantly positive effect of co-occurrence of ZIKV viral strain and *Ae*. *albopictus* population on the competence of *Ae*. *albopictus* for ZIKV was expected, however, none of the models in this meta-analysis support this. The lack of correlation may reflect a lack of coevolution between ZIKV and *Ae*. *albopictus* due to ZIKV’s relatively recent emergence, or it may be due to the broadness of our geographic characterizations (continent scale) or to a small sample size.

Analyses of both IR and TR at 7 and 14 dpi suggest that East Asian/Oceanic populations of *Ae*. *albopictus* have the greatest capacity to carry and transmit ZIKV (Fig 2). This may be because East Asian/Oceanic *Ae*. *albopictus* populations would have been the first to be infected by ZIKV as the virus spread from Africa eastward, thus giving the virus more time to adapt to its host in this part of the world [81]. However, as of yet, no field studies exist that examine the role of *Ae*. *albopictus* in ZIKV transmission in Asia [22]. The greater capacity of East Asian *Ae*. *albopictus* to carry and transmit ZIKV may have direct public health ramifications, as there is evidence that *Ae*. *albopictus* may already be the primary vector of DENV and CHIKV in some parts of Asia [82]. In these areas it seems likely that in the event of an outbreak, *Ae*. *albopictus* will also act as the primary vector for ZIKV, and thus vector control strategies should include *Ae*. *albopictus*. Furthermore, *Ae*. *albopictus’* ability to carry and transmit ZIKV highlights the need to understand its distribution, making vector surveillance of the species a high priority everywhere the species does or may occur.

Test of moderators for TR at 14 dpi also suggests that African strains of ZIKV have the highest potential to infect *Ae*. *albopictus* (Fig 3), which is in line with similar studies carried out on *Aedes aegypti* [53]. This study indicates a need to determine the potential of these strains to spread to areas with large populations of *Ae*. *albopictus*, such as Europe, North America, and Asia.

We expected to see a significant positive effect of fresh blood meals (vs. previously frozen blood meals) on *Ae*. *albopictus* TR, as described in previous papers [51,52], which we did not observe. Nor did we observe a significant difference between mosquitoes infected by feeding from live, murine blood meals and mosquitoes infected with artificial blood meals. This may be because of collinearity between fresh vs. frozen blood meal categories and the artificial vs. live blood meal categories in this study, or because of small sample size. The lack of effect of viral titer on IRs suggests that likelihood of infection for *Ae*. *albopictus* is not increased above a certain threshold, possibly around 1 x 10^7^ pfu/ffu/TCID_50_. These results demonstrate the necessity of evaluating the methods by which vector competence studies are carried out and creating a consensus regarding best practices for evaluating vector competence. This standardization could help to increase the speed with which the scientific community identifies vectors of emerging diseases and thus allow us to create effective vector control strategies in a timely manner.

There are also possible ecological barriers to *Ae*. *albopictus’* potential as a critical vector for ZIKV. Unlike the primary vector of ZIKV, *Ae*. *aegypti*, *Ae*. *albopictus* tends to take one large bloodmeal as opposed to several smaller ones, which limits *Ae*. *albopictus’* ability to transfer viruses from one organism to another [83]. *Ae*. *albopictus* has also been shown to be opportunistic in its feeding behaviors, taking blood meals from a wider variety of animals than the largely anthropophilic *Ae*. *aegypti*, though *Ae*. *albopictus’* blood meal preference seems to somewhat vary geographically [84–88]. More variable blood meal preference may make *Ae*. *albopictus* a less efficient vector than *Ae*. *aegypti* for ZIKV and other viruses, since *Ae*. *albopictus* are less likely to feed on humans than are *Ae*. *aegypti*. However, opportunistic feeding may also make *Ae*. *albopictus* a better bridge vector, carrying diseases such as ZIKV between humans and wildlife reservoirs, thus increasing its potential importance as a vector [37].

### Strengths and limitations

ZIKV was not commonly considered a major threat to public health before the 2015–2017 epidemic in the Americas. Therefore at the time of this study, relatively few studies of vector competence have been carried out on the disease, and most of them were done within a short time frame (Summer 2016 –Winter 2017). With an increasing number of studies, higher resolution analysis can be conducted that may provide insight into how ZIKV is evolving and adapting to new hosts and new environments. The high level of unexplained heterogeneity, as well as collinearity between variables in studies currently available may be due to small sample size (especially for TR at 7 dpi), exclusion criteria (for instance, exclusion of studies using viral titers below 1 x 10^7^ pfu/ffu/TCID_50_), or the influence of variables we did not consider in our models. Furthermore, considering the potential significance of a salivary gland barrier to transmission, this study’s inclusion of studies that measured TRs by the salivary glands instead of expectorated saliva may contribute to inflated estimates of TR, as there is potentially a salivary gland barrier that prevents viral expectoration in some mosquitoes [53,64]. Hopefully, future studies will allow re-examination the moderators in this meta-analysis in more detail and to evaluate their significance more robustly. Additionally, future field studies and virus/ vector population level studies will be important to validating the results of laboratory tests and to understand how outbreaks occur on a local level.

## Conclusion

The results of our study indicate that *Ae*. *albopictus* has the potential and competence to be a vector of ZIKV across the globe. The identification of *Ae*. *albopictus* as a competent vector for ZIKV raises multiple other points of concern, such as the continuation of *Ae*. *albopictus’* spread across North America and Europe [29,46], the high densities in which *Ae*. *albopictus* establishes itself [37,39], and the increasing realization that *Ae*. *albopictus* may either be growing more anthropophilic or may have always been more anthropophilic than previously thought [37,44]. Questions of vector competence are typically difficult to resolve given differing laboratory conditions and pathogen and vector sources. Here, we highlight the underutilized method of meta-analyses to clarify issues of vector competence studies, such as variable methodology which complicates comparison, given that this method has the potential to synthesize findings from existing studies, and unlike any individual study, allows researchers to incorporate a wide range of moderators to allow for better comparison between studies. Efforts to understand the ecology of both ZIKV and *Ae*. *albopictus* must be strengthened to understand what other, non-physiological, factors may still prevent *Ae*. *albopictus* from acting as a major vector for the disease. Finally, efforts at rural and sylvatic surveillance for ZIKV should increase. If *Ae*. *albopictus* can be infected by, and transmit ZIKV, there is more potential for the disease to become established in areas where monitoring systems are not looking for it.

## Supporting information

S1 Fig Funnel plots tended to have an asymmetrical shape, with the exception of the funnel for TR at 14 dpi (S1 Fig). Egger’s tests likewise suggested significant asymmetry for all funnel plots except for TR at 14 dpi (for IR at 7 dpi, P < 0.0012; for IR at 14 dpi, P <0.0001; for TR at 7 dpi, P < 0.0001; for TR at 14 dpi, P = 0.23).
(TIFF)

S1 Table Data used in the meta-analysis presented here. (DOCX)

S1 Checklist PRISMA checklist. (DOC)

S1 Data Infection and transmission rates. (XLSX)
