# Supplementary figures and images for: Aedes albopictus is a competent vector of Zika virus: A meta-analysis
Source: PLoS One. 2019 May 21;14(5):e0216794. doi: 10.1371/journal.pone.0216794 (PMC6528984; doi:10.1371/journal.pone.0216794)

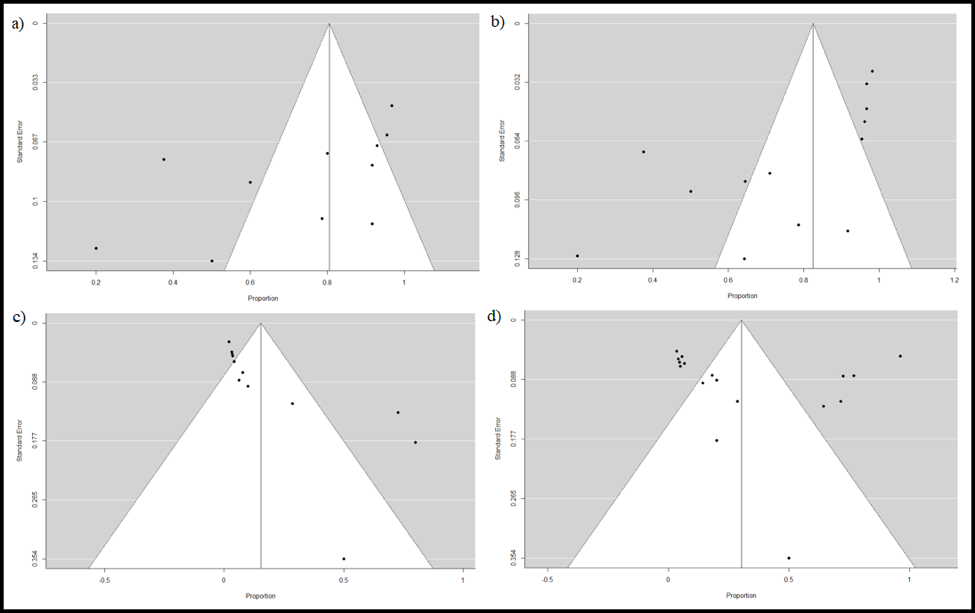

Supplement: S1 Fig — Egger’s tests likewise suggested significant asymmetry for all funnel plots except for TR at 14 dpi (for IR at 7 dpi, P < 0.0012; for IR at 14 dpi, P <0.0001; for TR at 7 dpi, P < 0.0001; for TR at 14 dpi, P = 0.23). (TIFF) [file pone.0216794.s001.tiff]
