# Supplementary material for: Aedes albopictus is a competent vector of Zika virus: A meta-analysis
Source: PLoS One. 2019 May 21;14(5):e0216794. doi: 10.1371/journal.pone.0216794 (PMC6528984; doi:10.1371/journal.pone.0216794)
Supplement: S1 Table — (DOCX) [file pone.0216794.s002.docx]

| Study | Origin of *Ae. albopictus* | Origin of Viral Strain | Geographic Co-occurrence | Virus frozen or fresh | Models in which study was used |
| --- | --- | --- | --- | --- | --- |
| Azar et al. 1 | North America | East Asia/ Oceania | No | Frozen | IR 7 dpi, IR 14 dpi, TR 7 dpi, TR 14dpi |
| Azar et al. 2 | North America | East Asia/ Oceania | No | Frozen | IR 7 dpi, IR 14 dpi, TR 7 dpi, TR 14dpi |
| Azar et al. 3 | North America | East Asia/ Oceania | No | Frozen | IR 7 dpi, IR 14 dpi, TR 7 dpi, TR 14dpi |
| Azar et al. 4 | North America | North America | Yes | Frozen | IR 7 dpi, IR 14 dpi, TR 7 dpi, TR 14dpi |
| Azar et al. 5 | North America | North America | Yes | Frozen | IR 7 dpi, IR 14 dpi, TR 7 dpi, TR 14dpi |
| Azar et al. 6 | North America | North America | Yes | Frozen | IR 7 dpi, IR 14 dpi, TR 7 dpi, TR 14dpi |
| Azar et al. 7 | North America | Africa | No | Frozen | IR 7 dpi, IR 14 dpi, TR 7 dpi, TR 14dpi |
| Azar et al. 8 | North America | Africa | No | Frozen | IR 7 dpi, IR 14 dpi, TR 7 dpi, TR 14dpi |
| Azar et al. 9 | North America | Africa | No | Frozen | IR 7 dpi, IR 14 dpi, TR 7 dpi, TR 14dpi |
| Azar et al. 10 | North America | South America | No | Frozen | IR 7 dpi, IR 14 dpi, TR 7 dpi, TR 14dpi |
| Azar et al. 11 | North America | South America | No | Frozen | IR 7 dpi, IR 14 dpi, TR 7 dpi, TR 14dpi |
| Azar et al. 12 | North America | North America | Yes | Frozen | IR 7 dpi, IR 14 dpi, TR 7 dpi, TR 14dpi |
| Azar et al. 13 | North America | North America | Yes | Frozen | IR 7 dpi, IR 14 dpi, TR 7 dpi, TR 14dpi |
| Azar et al. 14 | North America | South America | No | Frozen | IR 7 dpi, IR 14 dpi, TR 7 dpi, TR 14dpi |
| Azar et al. 15 | North America | South America | No | Frozen | IR 7 dpi, IR 14 dpi, TR 7 dpi, TR 14dpi |
| Azar et al. 16 | South America | South America | Yes | Frozen | IR 7 dpi, IR 14 dpi, TR 7 dpi, TR 14dpi |
| Azar et al. 17 | South America | South America | Yes | Frozen | IR 7 dpi, IR 14 dpi, TR 7 dpi, TR 14dpi |
| Azar et al. 18 | South America | North America | No | Frozen | IR 7 dpi, IR 14 dpi, TR 7 dpi, TR 14dpi |
| Azar et al. 19 | South America | North America | No | Frozen | IR 7 dpi, IR 14 dpi, TR 7 dpi, TR 14dpi |
| Chouin Carniero et al | North America | East Asia/ Oceania | No | Frozen | IR 14 dpi, TR 14dpi |
| DiLuca et al | Europe | East Asia/ Oceania | No | Fresh | IR 7 dpi, IR 14 dpi, TR 7 dpi, TR 14dpi |
| Duchemin et al | East Asia/ Oceania | East Asia/ Oceania | Yes | Fresh | IR 14 dpi, TR 14dpi |
| Garcia-Luna et al | North America | North America | Yes | Fresh | IR 7 dpi, IR 14 dpi, TR 7 dpi, TR 14dpi |
| Jupille et al | Europe | East Asia/ Oceania | No | Fresh | IR 7 dpi, IR 14 dpi, TR 7 dpi, TR 14dpi |
| Knecht et al | North America | North America | Yes | Fresh | IR 7 dpi |
| Heitmann et al 1 | Europe | North America | No | Fresh | IR 14 dpi, TR 14dpi |
| Heitmann et al 2 | Europe | North America | No | Fresh | IR 14 dpi, TR 14dpi |
| Liu et al | East Asia/ Oceania | East Asia/ Oceania | Yes | Frozen | IR 7 dpi, IR 14 dpi, TR 7 dpi, TR 14dpi |
| Lozano Fuentes et al | North America | North America | Yes | Fresh | IR 14 dpi, TR 14dpi |
| Richards et al | North America | South America | Yes | Fresh | IR 7 dpi, IR 14 dpi, TR 7 dpi, TR 14dpi |
| Ryckebusch et al | Europe | East Asia/ Oceania | No | Fresh | IR 7 dpi, IR 14 dpi, TR 7 dpi, TR 14dpi |
| Vazeille et al | East Asia/ Oceania | East Asia/ Oceania | Yes | Fresh | IR 7 dpi, IR 14 dpi |
| Wong et al | East Asia/ Oceania | Africa | No | Fresh | IR 7 dpi, IR 14 dpi, TR 7 dpi, TR 14dpi |
